# Minimal Dentinal Tubule Penetration of Endodontic Sealers in Warm Vertical Compaction by Direct Detection via SEM Analysis

**DOI:** 10.3390/jcm10194440

**Published:** 2021-09-27

**Authors:** Sina Schmidt, Edgar Schäfer, Sebastian Bürklein, Arno Rohrbach, David Donnermeyer

**Affiliations:** 1 Department of Periodontology and Operative Dentistry, Westphalian Wilhelms-University, Albert-Schweitzer-Campus 1, Building W 30, 48149 Münster, Germany; sina.schmidt@ukmuenster.de; 2 Central Interdisciplinary Ambulance in the School of Dentistry, Westphalian Wilhelms-University, Albert-Schweitzer-Campus 1, Building W 30, 48149 Münster, Germany; eschaef@uni-muenster.de (E.S.); sebastian.buerklein@ukmuenster.de (S.B.); 3 Institute for Mineralogy, Westphalian Wilhelms-University, Correnstraße 24, 48149 Münster, Germany; rohrbaa@uni-muenster.de

**Keywords:** AH Plus, endodontic sealer, scanning electron microscope, sealer penetration, Total Fill BC Sealer HiFlow

## Abstract

Sealer staining using rhodamine B dye to investigate the penetration depth of endodontic sealers was proven unsuitable for this purpose. This study aimed to investigate the sealer penetration depth into dentinal tubules by scanning electron microscopy (SEM). Root canals of 52 human upper central incisors were instrumented using the ProTaper Gold NiTi system (Dentsply Sirona, York, PA, USA) up to size F3. After irrigation with sodium hypochlorite and citric acid combined with ultrasonic activation, the root canals were either filled using the epoxy resin sealer AH Plus (Dentsply Sirona) or the calcium silicate-based sealer Total Fill BC Sealer HiFlow (TFHF, FKG Dentaire, La Chaux-de-Fonds, Switzerland) by warm vertical compaction. Root slices of 1 mm thickness were obtained at 2 to 3, 5 to 6 and 8 to 9 mm from the apex. The root slices were investigated for sealer penetration into the dentinal tubules using SEM according to four root quadrants (buccal, mesial, oral, distal). Statistical analysis was performed by the Kruskal-Wallis test (*p* = 0.05) as data were not normally distributed according to the Shapiro-Wilk test. AH Plus penetrated significantly deeper into the dentinal tubules compared to TFHF at each root level (*p* < 0.05). Dentinal sealer penetration was deeper in the bucco-oral direction compared to the mesio-distal direction. AH Plus penetrated deeper into dentinal tubules than TFHF. Warm vertical compaction exerting high pressure on the root canal filling material is not able to press sealers deep into dentinal tubules as penetration depth values did not exceed a mean of 110 µm in SEM.

## 1. Introduction

Warm vertical obturation of the root canal space was introduced to increase the amount of gutta-percha core material in a root canal filling and to use the pressure applied on the thermo-plasticized material to press endodontic sealer into isthmuses and dentinal tubules [1]. No other root canal obturation technique allows such forces to press sealer in the most unreachable parts of the root canal system [1]. 

Regarding sealer penetration depth into dentinal tubules, results inconsistent with the above-mentioned theorem have been published in the past. While some studies could not find a relationship between the filling technique and the penetration depth [2,3,4,5], a correlation of the penetration depth with the applied pressure on the root canal filling material was reported in one study [6]. Accordingly, though differences in tubule penetration are reasonable when different techniques are applied, no such effect can be concluded from the available studies. While it was argued that tooth-dependent factors such as variations of the root canal morphology could be the reason for the inconsistency of the studies [2], the results could also be interpreted as a lack of validation of the investigation technique [7]. Most studies used rhodamine B dye to stain the sealer. Lately, it was shown that this methodology is not able to indicate dentinal tubule penetration of a sealer but merely is capable of displaying the presence of dentinal tubule in a root slice [8]. As this is also an explanation for the variety of results presented in such studies on tubule penetration depth, the whole concept of a sealer penetrating deep into the root dentin tubules to ensure a tight seal of the root canal system was questioned.

No reliable data exist about the penetration depth of endodontic sealers others than from studies where sealers were labelled with rhodamine B [7,8]. Studies using a calcium marker indicated that depth of apatite crystal precipitation in the dentinal tubules is more inferior than expected from previous studies [4,7,9]. However, this technique is also not yet validated, and no method is available to evaluate the sealer penetration by direct detection of the sealer in the dentinal tubules underneath the specimen’s surface. Deepest penetration can be assumed when high pressure is applied on a softened root canal filling core material. Not all sealers are recommended to be used with warm vertical obturation techniques. Epoxy resin-based sealers such as AH Plus (Dentsply Sirona, York, PA, USA) are widely used in endodontics [10] and suitable for warm obturation [11]. Additionally, a new calcium silicate-based sealer, Total Fill BC Sealer HiFlow (TFHF) (FKG Dentaire, St. Maur de Fossés, Switzerland), was recently introduced [12]. TFHF is the first calcium silicate-based sealer that is recommended for warm vertical obturation, and from the yet-available data, it can be regarded safe to use for this purpose [13,14]. 

The present study was hence designed to investigate penetration depth of epoxy resin and calcium silicate-based sealers recommended for warm vertical root canal obturation into dentinal tubules. The investigation was performed using scanning electron microscopy as sealer staining using fluorescent dyes and was shown to be unsuitable to precisely indicate sealer penetration depth. The null hypothesis tested was that penetration depth would not differ between epoxy resin and calcium silicate-based sealers.

## 2. Materials and Methods

Sample size calculation was performed using G*Power (Heinrich Heine University, Düsseldorf, Germany) and indicated a group size of at least 26 samples per group (f = 0.4; α = 0.05, 1-β = 0.8). Thus, 52 freshly extracted central upper incisors were selected for the study and randomly divided into two experimental groups. All subjects gave their informed consent for inclusion of their extracted teeth. The study was conducted in accordance with the Declaration of Helsinki.

Prior to root canal treatment, all teeth were investigated under a stereomicroscope (Expert DN, Müller Optronic, Erfurt, Germany) to exclude cracks or root resorption. Buccal and proximal radiographs were taken to ensure a single root canal with an intact apical region and a single apical foramen. All teeth were cut coronally to a total length of 20 mm and access cavities were prepared when necessary. Apical patency of the root canal was checked using K-Files #10 and 15, and a working length of 19 mm was established. All root canals were instrumented using the ProTaper Gold NiTi system (Dentsply Sirona, York, PA, USA) up to size F3. The root canal was irrigated with 2.5 ml NaOCl 3% after each file and patency was checked after each instrument. After instrumentation, all root canals were irrigated twice with 2.5 mL NaOCl 3% combined with 30 s of ultrasonic activation (Irri-S, VDW.Ultra 30%-setting, VDW, Munich, Germany), 5 ml EDTA 17% and a final irrigation with 5 mL NaCl 0.9%. The roots were placed into plastic vials (Sarstedt, Numbrecht, Germany) and embedded into alginate (HenrySchein, Melville, NY, USA) as described in a previous study [15] to simulate temperature dissipation into tissues during warm vertical compaction. All roots were stored in an incubator (Memmert, Schwabach, Germany) at 37 °C and 100% humidity for 24 h before root canal obturation to ensure a specimen temperature of 37 °C. All root canal obturation procedures were performed in an incubator under this 37 °C environment as described previously [15]. Root canal obturation was performed according to the continuous wave technique using the 40/.06 heat plugger of the BeeFill obturation system (VDW) at a temperature setting of 200 °C and warm backfill. Prior to obturation, the root canals were dried using sterile paper points and either AH Plus or TFHF were inserted into the root canal using a matching F3 gutta-percha cone. After root canal obturation, the access cavities were sealed using an adhesive (OptiBond FL, Kerr, Rastatt, Germany) and composite resin (Estellite Sigma Quick Flow A3, Tokuyama, Tokyo, Japan). Afterwards, the roots were stored in an incubator for 8 weeks to ensure setting of the sealers. After incubation, the roots were removed from the alginate-filled vials and embedded horizontally into resin (Technovit 4071, Hereaus Kulzer, Hanau, Germany) after the buccal and mesial aspect of the tooth were marked and root slices with a thickness of 1 mm were cut at 2 to 3, 5 to 6 and 8 to 9 mm from the apex using a water-cooled diamond saw (Leitz, Wetzlar, Germany). To remove any smear layer, the specimens were rinsed with EDTA 17% and distilled water for 15 s each and exsiccated in a closed container afterward. Similar to other study protocols found in the literature, no ultrasonic irrigation was performed to avoid artefacts by removing sealer from the tubules [4,16,17]. The specimens’ apical surfaces were sputtered with gold, and sealer penetration was evaluated using scanning electron microscope (JSM 6510 LA, JEOL, Freising, Germany). The root sections were divided into four quadrants (buccal, mesial, oral, distal), and images were taken of each quadrant at 350× magnification. The materials pressed into the dentinal tubules were identified by EDX analysis as the above-mentioned sealers contain zirconium oxide as a radiopacifier. The highest penetration depth of sealer was recorded for each quadrant and statistical analysis was performed by the Kruskal–Wallis test (*p* = 0.05) as data were not normally distributed according to the Shapiro–Wilk test. 

## 3. Results

Regarding the mean penetration at each level, AH Plus penetrated significantly deeper into the dentinal tubules compared to TFHF (*p* < 0.05) (Table 1).

**Figure 1:**
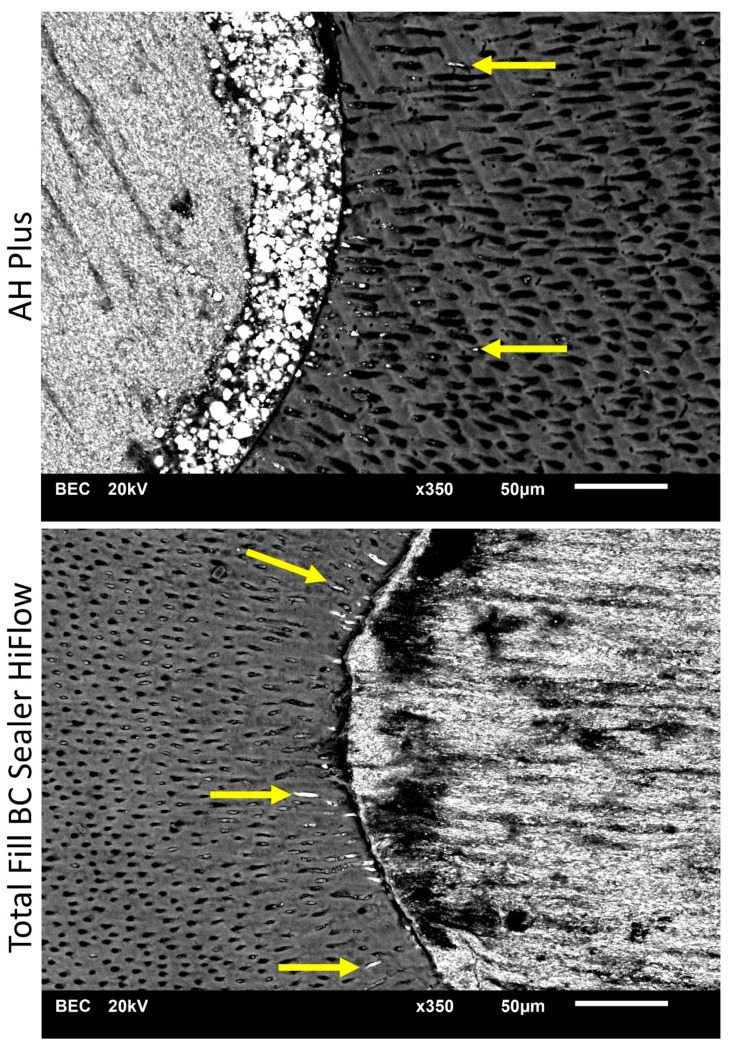
Scanning electron microscope (SEM) images display sealer penetration depth of AH Plus and Total Fill BC Sealer HiFlow after warm vertical root canal obturation at 2 mm from the root apex. Arrows indicating the sealer penetration depth found in SEM images.

**Figure 2:**
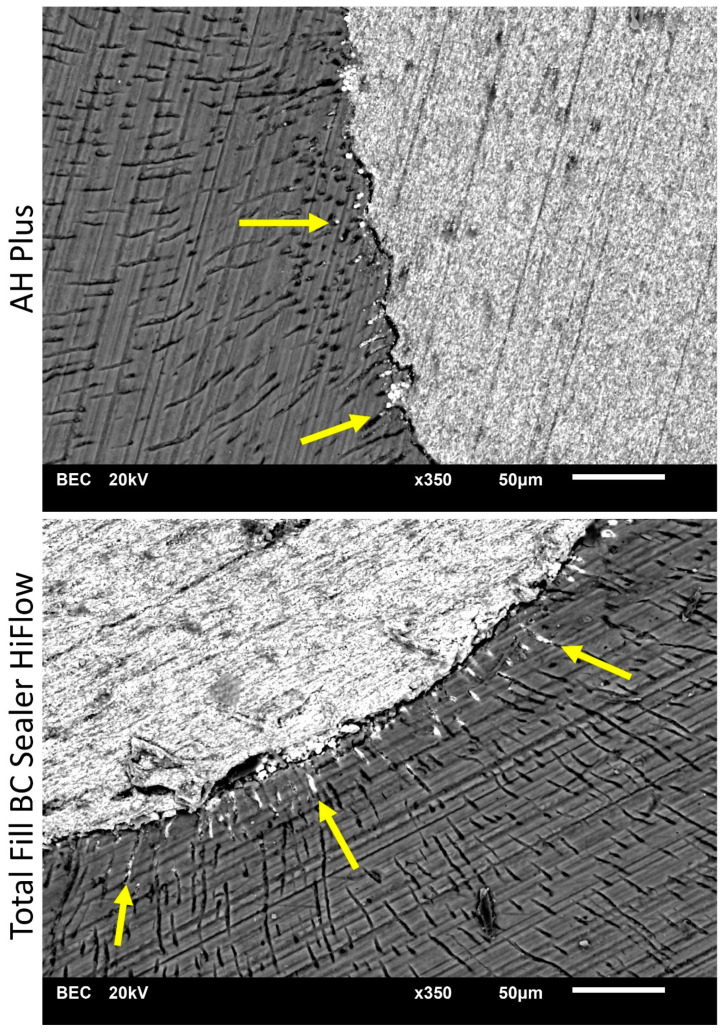
Scanning electron microscope (SEM) images display sealer penetration depth of AH Plus and Total Fill BC Sealer HiFlow after warm vertical root canal obturation at 5 mm from the root apex. Arrows indicating the sealer penetration depth found in SEM images.

**Figure 3:**
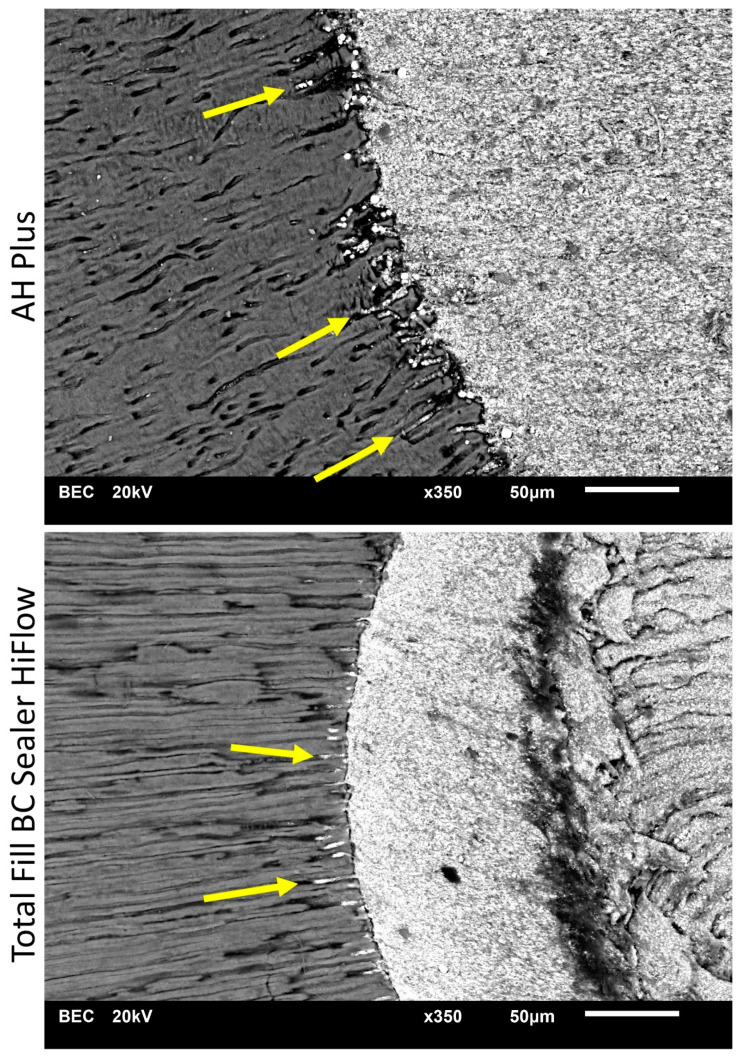
Scanning electron microscope (SEM) images display sealer penetration depth of AH Plus and Total Fill BC Sealer HiFlow after warm vertical root canal obturation at 8 mm from the root apex. Arrows indicating the sealer penetration depth found in SEM images.

In the apical portion of the root, AH Plus exhibited significantly deeper penetration than TFHF in the buccal quadrant (*p* < 0.05), while no statistical difference occurred in the other quadrants (*p* < 0.05). No statistical difference was found between AH Plus and TFHF according to the quadrants in the middle portion (*p* > 0.05). In the coronal portion, AH Plus showed significantly deeper penetration in the buccal, oral and distal section (*p* < 0.05) (Table 2). At all levels, dentinal penetration was deeper in the bucco-oral direction compared to the mesio-distal direction. Examples are given in Figure 1, Figure 2 and Figure 3.

## 4. Discussion

The penetration depth of endodontic sealers was evaluated by SEM investigation when a warm vertical compaction technique was used. AH Plus was found to penetrate deeper into dentinal tubules than TFHF in parts of the apical and coronal portion of the root. Therefore, the null hypothesis was rejected.

Sealer penetration depth is commonly investigated using CLSM. Sealers are mixed with organic dyes such as rhodamine B and indirectly detected by fluorescence of the organic dye [2]. It has been demonstrated that penetration depth of rhodamine B dye does not correlate with the detection of the sealer in the dentinal tubules by SEM [8]. According to the results of a SEM analysis, low penetration depth of the sealers resulted when a single cone technique was applied [8]. In this study, an attempt was made to increase the pressure on the sealer by warm vertical compaction to enforce sealer penetration in this study. Again, we tried to verify the sealer directly by SEM to avoid unreliable methods or confounding factors due to indirect display of the sealer. A major disadvantage, as mentioned before, is that SEM only allows sealer detection at the surface of the specimen, and, therefore, specimen preparation such as sawing could influence the results as sealer could be washed out of the tubules. In the present study, sealer penetration was detected only close to the main root canal and no single sealer spots without relation to the main root canal were found along the specimens during SEM investigation. Thus, a loss of sealer out of the tubules is unlikely. However, the method is limited to the specimen’s surface and can display only a small proportion of all dentinal tubules of the root dentine.

Overall, AH Plus showed significantly higher penetration depth values than TFHF at all root levels investigated. According to the quadrants at each level, significant differences did not occur in all levels. Penetration depths in mesial and distal quadrants were generally lower than in buccal or oral quadrants, which correlate with the number and size of the dentinal tubules [18]. In case differences were significant, AH Plus displayed deeper penetration into the dentinal tubules than TFHF. The deeper penetration of AH Plus can be explained by the behaviour of the two sealer types investigated during root canal obturation, especially when heat is applied. Heat application resulted in a decreased viscosity of TFHF [14]. This could influence the ability to penetrate dentinal tubules when heat is applied. A reduction of temperature and the use of low-melting gutta-percha could influence the viscosity positively, but deep penetration remains unlikely. However, particle size of the sealer seems not to have a significant effect on penetration. In theory, it is more likely that smaller particles are pressed more deeply into the tubules when the same pressure is applied. According to the results, no such phenomenon was observed. A particle size of up to 8 µm was reported for AH Plus. No such date exists for TFHF, but for the predecessor product Total Fill BC Sealer (FKG Dentaire) a particle size of less than 0.2 µm is given [19]. Both sealers have been found suitable for warm vertical obturation in laboratory experiments so far but might show a different behaviour under clinical conditions [11,14]. Therefore, viscosity and the material characteristics after heat application seem to be favourable for deeper penetration when an epoxy resin is used.

**Figure 4:**
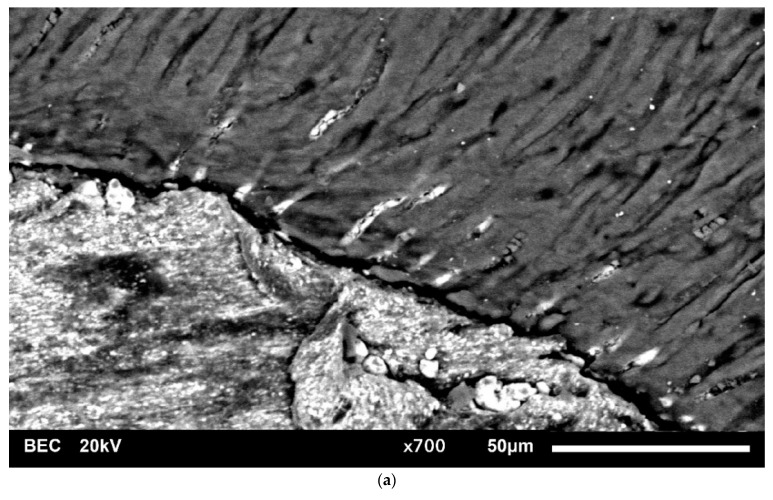

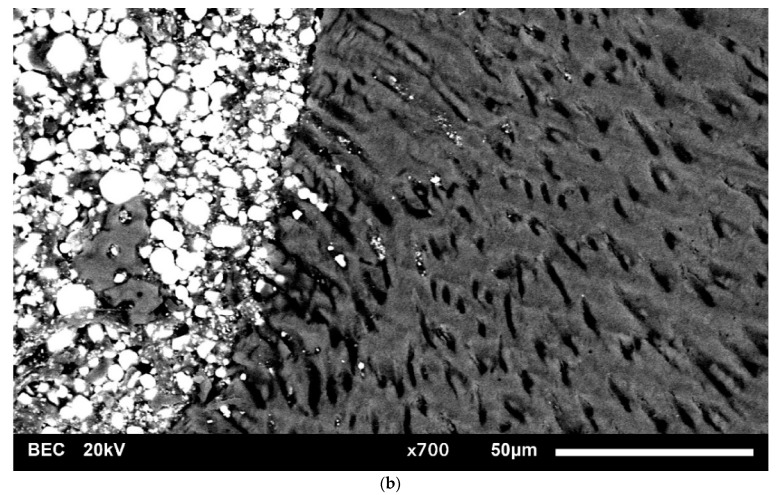
High magnification SEM images (×700) of the sealer-dentin-interface. (**a**) showing low extent of TFHF penetration into dentinal tubules and (**b**) showing low extent of AH Plus penetration into dentinal tubules.

Comparing the present results to previous studies, it is apparent that penetration depth was found only in a small extent (Figure 4). Penetration depth mean values with a maximum of 108.15 ± 56.42 µm (AH Plus, coronal part, buccal section) were observed whereas other studies reported penetration of up to 2000 µm [16,17,20]. It is apparent that the methodology is the main reason for the disparity of results [8]. According to a previously published study [8], it seems likely that staining of sealers using fluorescent dyes like rhodamine B is not a reliable method to investigate sealer penetration. Moreover, sealer penetration into dentinal tubules might have been overestimated in the past and is merely a marginal effect during root canal obturation [8]. Additionally, most studies only evaluate maximum penetration values while penetration occurs in specific patterns depending on the size and number of dentinal tubules [4,16,17,21]. In most parts of a specimen the dentinal tubule penetration is lower than the values used for statistical analysis. The thesis of overestimated dentinal tubule penetration is supported by recent findings that dentinal tubule penetration investigated by CLSM did not correlate with dislodgement resistance of root canal fillings [22].

Sealer penetration deep into dentinal tubules is implausible according to the results of the present study. Though the penetration of fluorescent dyes that were added to the sealer in several studies was reported [16,20,23] no correlation was found between dye penetration and direct sealer verification [8]. Three dimensional or other methods able to investigate the sealer penetration underneath the specimen’s surface must be developed to reliably evaluate the presence of sealer in dentinal tubules. As so far, penetration of endodontic sealer deep into dentinal tubules was not directly verified.

## 5. Conclusions

Penetration of endodontic sealers deep into dentinal tubules of the root dentin is unlikely. Even high pressure on the root canal filling material is not able to press sealer deep into dentinal tubules. AH Plus penetrated deeper into dentinal tubules than TFHF in parts of the apical and coronal portion of the root.

## Figures and Tables

**Table 1:** Means and standard deviations for sealer penetration (µm) of AH Plus and Total Fill BC Sealer HiFlow in the apical, middle and coronal portion of the root.

Sealer	Apical	Middle	Coronal
AH Plus	49.45 ± 56.40 ^A^	43.69 ± 49.64 ^A^	87.57 ± 58.31 ^A^
Total Fill BC Sealer HiFlow	24.15 ± 36.11 ^B^	18.33 ± 14.19 ^B^	33.06 ± 30.11 ^B^

Values with different superscript letters in columns indicate statistical differences at *p* = 0.05 (Kruskal–Wallis test).

**Table 2:** Means and standard deviations for sealer penetration (µm) of AH Plus and Total Fill BC Sealer HiFlow in the apical, middle and coronal portion of the root according to the root quadrant.

		AH Plus	Total Fill BC Sealer HiFlow
apical	buccal	59.19 ± 66.71 ^A^	10.71 ± 38.72 ^B^
	mesial	35.75 ± 41.12 ^A^	28.38 ± 39.76 ^A^
	oral	53.45 ± 63.47 ^A^	35.13 ± 37.34 ^A^
	distal	49.41 ± 51.26 ^A^	22.37 ± 38.72 ^A^
middle	buccal	60.37 ± 58.27 ^A^	22.84 ± 16.82 ^A^
	mesial	16.33 ± 25.75 ^A^	14.53 ± 11.83 ^A^
	oral	63.30 ± 56.45 ^A^	20.84 ± 11.58 ^A^
	distal	34.75 ± 37.03 ^A^	15.08 ± 14.78 ^A^
coronal	buccal	108.15 ± 56.42 ^A^	25.71 ± 21.70 ^B^
	mesial	69.80 ± 47.42 ^A^	34.01 ± 25.13 ^A^
	oral	88.22 ± 74.80 ^A^	33.60 ± 34.09 ^B^
	distal	84.10 ± 46.88 ^A^	38.91 ± 37.12 ^B^

Values with different superscript letters in rows indicate statistical differences at *p* = 0.05 (Kruskal–Wallis test).

## Data Availability

The data presented in this study are available on request from the corresponding author.

## References

[1] Schilder H., Hargreaves K.M. (2006). Filling root canals in three dimensions. J. Endod..

[2] Reynolds J.Z., Augsburger R.A., Svoboda K.K.H., Jalali P. (2020). Comparing dentinal tubule penetration of conventional and “HiFlow” bioceramic sealers with resin-based sealer: An in vitro study. Aust. Endod. J..

[3] Weis M.V., Parashos P., Messer H.H. (2004). Effect of obturation technique on sealer cement thickness and dentinal tubule penetration. Int. Endod. J..

[4] Jeong J.W., DeGraft-Johnson A., Dorn S.O., Di Fiore P.M. (2017). Dentinal Tubule Penetration of a Calcium Silicate–based Root Canal Sealer with Different Obturation Methods. J. Endod..

[5] Wang Y., Liu S., Dong Y. (2018). In vitro study of dentinal tubule penetration and filling quality of bioceramic sealer. PLoS ONE.

[6] De Deus G.A., Gurgel-Filho E.D., Maniglia-Ferreira O., Coutinho-Filho T. (2004). The influence of filling technique on depth of tubule penetration by root canal sealer: A study using light microscopy and digital image processing. Aust. Endod. J..

[7] Furtado T.C., de Bem I.A., Machado L.S., Pereira J.R., Reis Só M.V., da Rosa R.A. (2021). Intratubular penetration of endodontic sealers depends on the fluorophore used for CLSM assessment. Microsc. Res. Tec..

[8] Donnermeyer D., Schmidt S., Rohrbach A., Bürklein S., Schäfer E. (2021). Debunking the concept of dentinal tubule penetration of endodontic sealers—Sealer staining with rhodamine B fluorescent dye is an inadequate method. Materials.

[9] Coronas V.S., Villa N., do Nascimento A.L., Duarte P.H.M., da Rosa R.A., Reis Só M.V. (2020). Dentinal tubule penetration of a calcium silicate-based root canal sealer using a specific calcium fluorophore. Braz. Dent. J..

[10] Hergt A., Wiegand A., Hülsman M., Rödig T. (2015). AH Plus root canal sealer-an updated literature review. Endo.

[11] Donnermeyer D., Urban K., Bürklein S., Schäfer E. (2020). Physico-chemical investigation of endodontic sealers exposed to simulated intracanal heat application: Epoxy resins and zinc oxide–eugenols. Int. Endod. J..

[12] Donnermeyer D., Dammaschke T., Schäfer E. (2020). Hydraulic calcium silicate-based sealers: A game changer in root canal obturation?. Endod. Pract. Today.

[13] Hadis M., Camilleri J. (2020). Characterization of heat resistant hydraulic sealer for warm vertical obturation. Dent. Mater..

[14] Chen B., Haapasalo M., Mobuchon C., Li X., Ma J., Schn Y. (2020). Cytotoxicity and the Effect of Temperature on Physical Properties and Chemical Composition of a New Calcium Silicate–based Root Canal Sealer. J. Endod..

[15] Donnermeyer D., Schäfer E., Bürklein S. (2018). Real-time Intracanal Temperature Measurement During Different Obturation Techniques. J. Endod..

[16] El Hachem R., Khalil I., le Brun G., Pellen F., Le Jeune B., Daou M., El Osta N., Naaman A., Abboud M. (2019). Dentinal tubule penetration of AH Plus, BC Sealer and a novel tricalcium silicate sealer: A confocal laser scanning microscopy study. Clin. Oral. Invest..

[17] McMichael G.E., Primus C.M., Opperman L.A. (2016). Dentinal tubule penetration of tricalcium silicate sealers. J. Endod..

[18] Schellenberg U., Krey G., Bosshardt D., Nair P.N. (1992). Numerical density of dentinal tubules at the pulpal wall of human permanent premolars and third molars. J. Endod..

[19] Asawaworarit W., Pinyosopon T., Kijsamanmith K. (2020). Comparison of apical sealing ability of bioceramic sealer and epoxy resin-based sealer using the fluid filtration technique and scanning electron microscopy. J. Dent. Sci..

[20] Uzunoglu-Özyürek E., Erdoğan Ö., Aktemur Türker S. (2018). Effect of Calcium Hydroxide Dressing on the Dentinal Tubule Penetration of 2 Different Root Canal Sealers: A Confocal Laser Scanning Microscopic Study. J. Endod..

[21] Bolles J.A., He J., Svoboda K.K., Schneiderman E., Glickman G.N. (2013). Comparison of Vibringe, EndoActivator, and needle irrigation on sealer penetration in extracted human teeth. J. Endod..

[22] De-Deus G., Brandão M.C., Souza E.M., Reis C., Reis K., Machado R., Neelakantan P. (2017). Epoxy resin-based root canal sealer penetration into dentin tubules does not improve root filling dislodgement resistance. Eur. Endod. J..

[23] Aydın Z.U., Özyürek T., Keskin B., Baran T. (2019). Effect of chitosan nanoparticle, QMix, and EDTA on TotalFill BC sealers’ dentinal tubule penetration: A confocal laser scanning microscopy study. Odontology.

